# The role of acute normovolemic hemodilution in reducing allogeneic blood transfusion in glioblastoma surgery: a case–control study

**DOI:** 10.1186/s41016-023-00343-2

**Published:** 2023-11-13

**Authors:** Ping Chen, Xin-Huang Zhang, Ying Wang, Xian-Zhong Lin, De-Zhi Kang, Qing-Song Lin

**Affiliations:** 1 Department of Anesthesiology, The First Affiliated Hospital of Fujian Medical University, Fuzhou, 350005 Fujian China; 2 Department of Neurosurgery, The First Affiliated Hospital of Fujian Medical University, No. 20 Chazhong Rd, Taijiang District, Fuzhou, 350005 Fujian China; 3 Department of Neurosurgery, Binhai Branch of National Regional Medical Center, The First Affiliated Hospital of Fujian Medical University, Fuzhou, 350209 Fujian China; 4 Fujian Provincial Institutes of Brain Disorders and Brain Sciences, The First Affiliated Hospital of Fujian Medical University, Fuzhou, 350005 Fujian China; 5 Fujian Provincial Clinical Research Center for Neurological Diseases, The First Affiliated Hospital of Fujian Medical University, Fuzhou, 350005 Fujian China; 6 Fujian Provincial Key Laboratory of Precision Medicine for Cancer, The First Affiliated Hospital of Fujian Medical University, Fuzhou, 350005 Fujian China

**Keywords:** Glioblastoma, Acute normovolemic hemodilution, Surgery, Blood transfusion

## Abstract

**Background:**

Acute normovolemic hemodilution (ANH) was first introduced in glioblastoma surgery, and its role in reducing allogeneic blood transfusion was investigated in this study.

**Methods:**

This study enrolled supratentorial glioblastoma patients who received total resection. In the ANH group, the patients were required to draw blood before the operation, and the blood will be transfused back to the patient during the operation. The association between ANH and clinical features was investigated.

**Results:**

Sixty supratentorial glioblastoma patients were enrolled in this study, 25 patients were allocated in the ANH group, and another 35 patients were included in the control group. ANH dramatically reduced the need for allogeneic blood transfusion (3 [12%] vs 12 [34.3%], *P* = 0.049), and the blood transfusion per total of patients was dramatically decreased by the application of ANH (0.40 ± 1.15 units vs 1.06 ± 1.59 units, *P* = 0.069). Furthermore, ANH also markedly reduced the requirement of fresh frozen plasma (FFP) transfusion (2 [8%] vs 11 [31.4%], *P* = 0.030) and the volume of FFP transfusion per total of patients (32.00 ± 114.46 mL vs 115.71 ± 181.00 mL, *P* = 0.033). The complication rate was similar between the two groups.

**Conclusions:**

ANH was a safe and effective blood conservation technique in glioblastoma surgery.

## Background

Glioblastoma is the most common malignancy brain tumor [1]. Maximum safe resection is recommended as the first-step key treatment [2]. Major glioblastoma resection may encounter large blood loss and require red blood cell (RBC) transfusion and fresh frozen plasma (FFP) transfusion [3, 4]. As reported, 48.9% of patients who underwent glioblastoma surgery received allogeneic blood transfusion [4]. Allogeneic blood transfusion for patients who underwent cranial surgery is associated with substantive postoperative mortality and morbidity [5]. However, preparation of sufficient blood product is important to ensure the safety of glioblastoma surgery. Nevertheless, blood supply was often insufficient in clinical practice [6, 7]. Furthermore, allogeneic blood transfusion has some well-known disadvantages, including immunomodulatory effects which may affect innate immunity to suppress the growth and spread of the tumor [8, 9]. Therefore, it is crucial to reduce allogeneic blood transfusion.

Acute normovolemic hemodilution (ANH) is a clinical technique for blood conservation. ANH collects autologous blood immediately before the surgery and maintains euvolemia with crystalloid/colloid replacement [10]. The harvested autologous blood will be returned to the patient before completing the surgery. ANH has been successfully used in hepatic, cardiac, and orthopedic surgeries, and ANH was suggested to reduce allogeneic blood transfusion, preserve coagulation factors, and eliminate the possibility of clerical error [11–13]. However, the role of ANH in glioblastoma surgery remains largely unknown.

This study aimed to assess the role of ANH in reducing perioperative allogeneic blood transfusion and identify the impact of ANH on patients’ outcomes in glioblastoma surgery.

## Methods

### Patient enrollment and study design

A case–control study was carried out in our hospital to identify the role of ANH in glioblastoma surgery. Adult patients older than 18 years with a pathological diagnosis of supratentorial glioblastoma who underwent gross total resection were enrolled. Patients with ANH were allocated to the ANH group, and the control group did not receive ANH. We excluded (1) hemoglobin < 10.0 g/dL or serum hematocrit < 28% preoperatively; (2) combined with systematic disease, including congestive heart disease, obstructive pulmonary disease, or abnormal coagulation status; (3) combined with cerebrovascular disease or other intracranial lesions; (4) preoperative blood donation or receiving erythropoietin; (5) tumor localized at brainstem, spinal cord, or cerebellum; (6) partial resection or biopsy; and (7) incomplete or missing data. The STROBE statement was followed in this study. This study was approved by the ethics committee of First Affiliated Hospital of Fujian Medical University. Informed consent was all obtained from the patients.

### Perioperative surgical management

The guidelines of the European Association for Neuro-Oncology (EANO) were obeyed in the management of glioblastoma [14]. Briefly, the goal of surgery is to safely remove as much tumor as possible. None of the patients in this cohort underwent preoperative supply vessel embolization. Gross total resection should yield the neurofunctional protection when tumor cells infiltrated the eloquent areas. All patients in the ANH group received 400 mL of whole blood withdrawn. The blood was collected through a radial arterial line and stored in a standard disposable plastic autologous blood collection bag (Shanghai Blood Transfusion Technology Co., Ltd., China) at room temperature. During collection, a tilt rocker scale (CZK-IB, Suzhou Medical Instrument Company, China) was applied to mix and assess the blood. Euvolemia was achieved by transfusing 600 mL crystalloid and 200 mL colloid. Perioperative blood transfusion followed the guidelines of the American Society of Anesthesiologists Task Force on Perioperative Blood Transfusion and Adjuvant Therapies [15]. A hemoglobin < 7.0 g/dL was set as the intraoperative transfusion trigger. Autologous blood was transfused to patients if the intraoperative transfusion trigger was met; if it did not reach the transfusion trigger or the surgery was performed for more than 8 h, the autologous blood was reinfused then. Only when autologous blood had been used, the allogeneic blood may be ordered. In the non-ANH group, allogeneic blood was ordered when the transfusion trigger was reached. Postoperatively, allogeneic blood was ordered when suffering hemodynamic changes or hemoglobin < 8 g/dL. FFP transfusion was indicated when encountered huge microvascular bleeding intraoperatively or INR > 1.8.

### Statistical analysis

The outcome was evaluated with the Karnofsky Performance Scale (KPS) at discharge, and the KPS scores were divided into favorable (KPS 70–100) and unfavorable (KPS 0–60) outcomes. Continuous variables were expressed as mean ± SD, and categorical variables were presented as counts with percentages. The continuous variables were assessed by independent sample *t* test or the Mantel–Haenszel test, and the categorical variables were measured by *χ*^2^ test or the Fisher’s exact test. The results with *P* < 0.05 were considered statistically significant. All the statistical analysis was conducted with the SPSS (IBM Statistic 20.0).

## Results

**Fig. 1:**
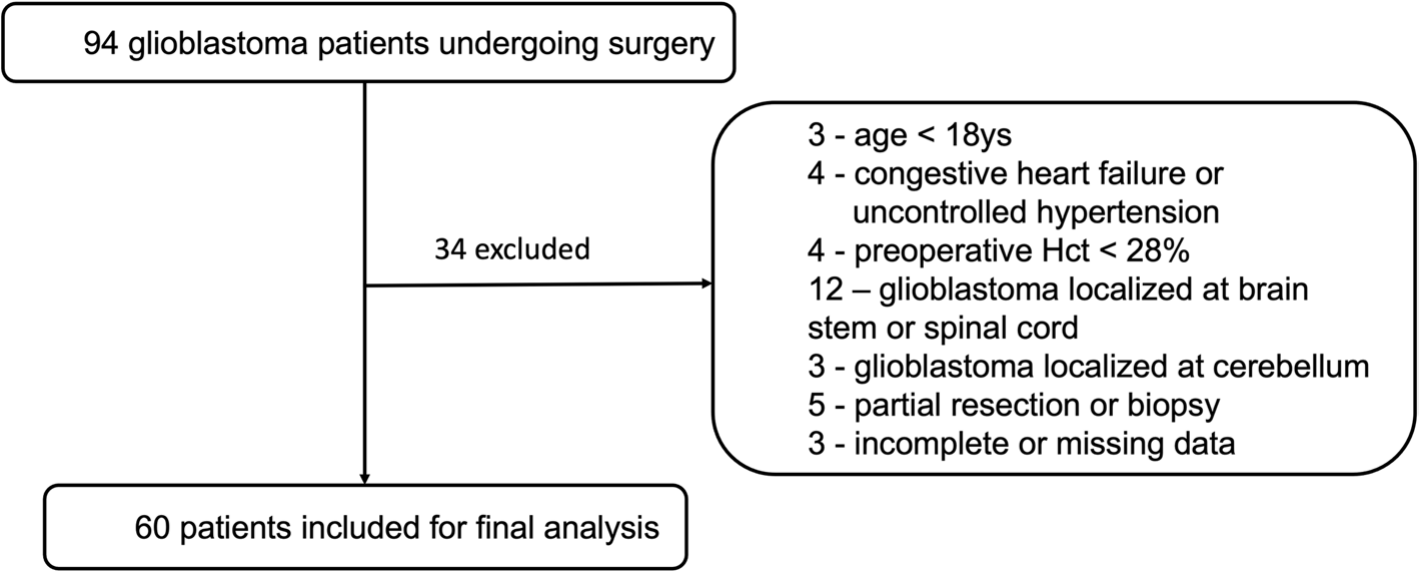
Flow chart showed the enrollment of glioblastoma patients in this study

A total of 94 patients who underwent glioblastoma surgery were identified in our hospital between Jan 2020 and Dec 2020. Sixty patients were included in the final analysis (Fig. 1), 25 in the ANH group, and 35 in the non-ANH group.

### Patient characteristics and surgical data

Patient characteristics were similar between the two groups (Table 1). The conduction of ANH had no obvious impact on the length of surgery (227.5 ± 73.2 min vs 252.8 ± 81.1 min, *P* = 0.219, Table 2). The amount of blood loss was similar between the two groups. There were no differences in the amount of total fluid given, the amount of crystalloid given, the amount of colloid given, and the intraoperative urine output (Table 2). Hemodynamics, including systolic blood pressure and central venous pressure, was stably maintained within the normal range throughout the operation in both groups.

**Table 1:** Comparison of demographic and preoperative data

	**ANH (*n* = 25)**	**Non-ANH (*n* = 35)**	***P* value**
Age (year)	48.5 ± 16.2	49.1 ± 13.9	0.873
Male, %	14 (56%)	24 (68.6%)	0.319
Body mass index	22.8 ± 2.8	23.7 ± 3.5	0.289
Hypertension, %	7 (28%)	6 (17.1%)	0.314
ASA classification, %			
1	3 (12%)	6 (17.1%)	0.863
2	21 (84%)	28 (80%)	
3	1 (4%)	1 (2.9%)	
Glioblastoma characteristics			
Left, %	8 (32%)	17 (48.6%)	0.199
Recurrence glioblastoma, %	3 (12%)	5 (14.3%)	0.797
Deep location, %	13 (52%)	12 (34.3%)	0.170
Tumor size (cm)	4.9 ± 1.7	5.0 ± 2.3	0.937
Preoperative laboratory studies			
Hemoglobin (g/dL)	13.9 ± 1.7	13.9 ± 1.4	0.967
Platelet count (K/μL)	248.4 ± 70.7	221.5 ± 57.0	0.107
INR	1.03 ± 0.09	1.00 ± 0.07	0.152
PT (s)	12.6 ± 1.1	12.1 ± 0.9	0.107
APTT (s)	32.8 ± 6.1	33.1 ± 5.7	0.880
BUN (mmol/L)	5.5 ± 2.2	5.0 ± 1.4	0.326
Bilirubin (mg/dL)	7.0 ± 2.6	8.0 ± 3.5	0.233
Albumin (g/dL)	41.7 ± 3.1	42.8 ± 3.9	0.242
Potassium (mmol/L)	4.2 ± 0.5	4.1 ± 0.4	0.205
Calcium (mmol/L)	2.3 ± 0.09	2.3 ± 0.1	0.411

Data are mean ± SD or *n* (%). *P* values were calculated by Student’s *t* test, Mann–Whitney *U* test, chi-square test, or Fisher’s exact test, as appropriate. *ANH* acute normovolemic hemodilution, *ASA classification* American Society of Anesthesiologists classification, *INR* international normalized ratio, *PT* prothrombin time, *APTT* activated partial thromboplastin time, *BUN* urea nitrogen

**Table 2:** Comparison of perioperative results

	**ANH (*n* = 25)**	**Non-ANH (*n* = 35)**	***P* value**
Length of surgery (min)	227.5 ± 73.2	252.8 ± 81.1	0.219
Estimated blood loss (mL)	388.0 ± 237.3	498.6 ± 392.7	0.181
Intraoperative fluid given			
Total (mL)	2556.0 ± 595.7	2726.6 ± 1179.3	0.466
Crystalloid (mL)	1504.0 ± 422.5	1401.4 ± 511.3	0.414
Colloid (mL)	864.0 ± 309.4	1008.6 ± 514.1	0.216
Intraoperative urine output (mL)	934.0 ± 486.2	805.7 ± 516.9	0.335
Postoperative laboratory studies (day 1)			
Hemoglobin (g/dL)	12.7 ± 1.9	11.6 ± 1.6	0.021
Platelet count (K/μL)	211.6 ± 58.4	193.2 ± 56.4	0.225
INR	1.03 ± 0.084	1.02 ± 0.064	0.673
PT (s)	11.9 ± 1.0	11.7 ± 0.7	0.476
APTT (s)	24.3 ± 5.6	25.8 ± 4.7	0.284
BUN (mmol/L)	5.3 ± 1.9	4.9 ± 1.6	0.370
Bilirubin (mg/dL)	6.9 ± 1.8	7.3 ± 3.4	0.594
Potassium (mmol/L)	4.13 ± 0.50	4.10 ± 0.44	0.764
Calcium (mmol/L)	2.15 ± 0.10	2.12 ± 0.14	0.451
Complications			
Hematoma, %	1 (4%)	2 (5.7%)	0.764
Intracranial infection, %	2 (8%)	4 (11.4%)	0.663
Pulmonary infection, %	4 (16%)	2 (5.7%)	0.383
Hydrocephalus	1 (4%)	2 (5.7%)	0.764
Hospital length of stay (day)	17.5 ± 6.9	17.3 ± 6.2	0.936
Clinical outcome at discharge			
Unfavorable outcome (KPS score < 70), %	8 (32%)	10 (28.6%)	0.775
Mortality, %	1 (4%)	1 (2.9%)	0.808

Data are mean ± SD or *n* (%). *P* values were calculated by Student’s *t* test, Mann–Whitney *U* test, chi-square test, or Fisher’s exact test, as appropriate. *ANH* acute normovolemic hemodilution, *INR* international normalized ratio, *PT* prothrombin time, *APTT* activated partial thromboplastin time, *BUN* urea nitrogen, *KPS score* Karnofsky Performance Scale

The complication rate was similar between the two groups. A total of 21 complications were identified in 19 (31.7%) patients in the entire cohort, 9 complications in 8 ANH patients, and 12 complications in 11 non-ANH patients (8 [32%] vs 11 [31.4%], *P* = 0.963). The complication rates were similar to previous reports (30.3–47.6%) [3, 16]. Pulmonary infection (*n* = 6, 10%) and intracranial infection (*n* = 6, 10%) were the two most common complications, both of which occurred similarly in ANH patients and in non-ANH patients (4 [16%] vs 2 [5.7%], *P* = 0.383; 2 [8%] vs 4 [11.4%], *P* = 0.663, respectively, Table 2). Three patients encountered postoperative intracranial hematoma and underwent a second surgery, 1 in the ANH group and 2 in the non-ANH group (1 [4%] vs 2 [5.7%], *P* = 0.764). Three patients developed hydrocephalus, 1 in the ANH group and 2 in the non-ANH group (1 [4%] vs 2 [5.7%], *P* = 0.764). Each group had 1 patient who suffered from severe brain edema. One patient in the non-ANH group experienced incision infection. No patients had myocardial ischemia or infarction. The discharge mortality rate has likewise no difference, with 1 death in each group. Hospital length of stay was similar between the two groups (Table 2). None of the complications was suggested related to ANH, which was reevaluated by two investigators.

### Perioperative routine laboratory analysis

The intraoperative hemoglobin levels at the point of completing autologous blood collection in the ANH group decreased sharply, but the difference in intraoperative hemoglobin levels was not significant between the two groups (12.9 ± 2.1 g/dL vs 13.6 ± 1.7 g/dL,* P* = 0.201, Table 3). On postoperative day 1, there was no difference in routine laboratory examinations, except the serum hemoglobin level, which was significantly higher in ANH patients than that in non-ANH patients (12.7 ± 1.9 g/dL vs 11.6 ± 1.6 g/dL, *P* = 0.021, Table 3). Compared to postoperative day 1, the average hemoglobin levels decreased at least 0.5 g/dL in both groups on postoperative day 3, and the difference remained significant with a higher level of hemoglobin in ANH patients (12.0 ± 1.6 g/dL vs 10.7 ± 1.4 g/dL, *P* = 0.002, Table 3). There was no difference in the postoperative circulating platelet count between the two groups (Table 3).

**Table 3:** Perioperative blood routine examination and transfusion data for all patients

	**ANH (*n* = 25)**	**Non-ANH (*n* = 35)**	***P* value**
Hemoglobin (g/dL)			
Preoperative	13.9 ± 1.7	13.9 ± 1.4	0.967
Intraoperative	12.9 ± 2.1	13.6 ± 1.7	0.201
Postoperative day 1	12.7 ± 1.9	11.6 ± 1.6	0.021
Postoperative day 3	12.0 ± 1.6	10.7 ± 1.4	0.002
Platelet count (K/μL)			
Preoperative	248.4 ± 70.7	221.5 ± 57.0	0.107
Postoperative day 1	211.6 ± 58.4	193.2 ± 56.4	0.225
Postoperative day 3	202.8 ± 63.1	180.0 ± 33.7	0.108
Allogeneic RBC transfusion			
Patients, %	3 (12%)	12 (34.3%)	0.049
Total volume (U)	6	37	
Unit per patient with transfusion (U)	3.33 ± 1.15	3.08 ± 1.00	0.711
Unit per total of patients (U)	0.40 ± 1.15	1.06 ± 1.59	0.069
FFP transfusion			
Patients, %	2 (8%)	11 (31.4%)	0.030
Total volume (mL)	800	4050	
Volume per patient with transfusion (mL)	400.00 ± 141.42	368.18 ± 95.58	0.689
Volume per total of patients (mL)	32.00 ± 114.46	115.71 ± 181.00	0.033

Data are mean ± SD. *P* values were calculated by Student’s *t* test, or Mann–Whitney *U* test, as appropriate. *ANH* acute normovolemic hemodilution, *RBC* red blood cell, *FFP* fresh frozen plasma

### Transfusion data

A total of 15 (20%) patients received intraoperative allogeneic RBC transfusion, 3 in the ANH group and 12 in the non-ANH group, and the rate of intraoperative allogeneic RBC transfusion in the ANH group was about 1/3 lower than that in the non-ANH group (3 [12%] vs 12 [34.3%], *P* = 0.049, Table 3). No patient received postoperative allogeneic blood transfusion. A sum of 43 units of RBC was used in the entire cohort, 6 units in the ANH group and 37 units in the non-ANH group. For patients who received allogeneic RBC transfusion, the average transfusion units have no difference between the two groups (3.33 ± 1.15 units vs 3.08 ± 1.00 units, *P* = 0.771, Table 3). While concerning the transfusion units per total of patients, ANH reduced about 2.5 times the units of allogeneic RBC transfusion per total of patients than that in the non-ANH group, although the difference did not reach significance (0.40 ± 1.15 units vs 1.06 ± 1.59 units, *P* = 0.069, Table 3). Eight patients in the ANH group returned the autologous blood intraoperatively because of the arrival of the transfusion trigger, and 5 of these patients did not require allogeneic RBC transfusion. Other patients in the ANH group reentered the autologous blood at the completion of surgery.

A total of 13 (21.7%) patients received FFP transfusion in the entire cohort, 2 in the ANH group and 11 in the non-ANH group, and the FFP transfusion rate was about 1/4 lower in the ANH group than that in the non-ANH group (2 [8%] vs 11 [31.4%], *P* = 0.030, Table 3). In total, 4850 mL of FFP was administered, 800 mL in ANH patients, and 4050 mL in non-ANH patients. For patients who received FFP transfusion, the volume of FFP transfusion per patient has no difference between the two groups (400.00 ± 141.42 mL vs 368.18 ± 95.58 mL, *P* = 0.689, Table 3). While comparing the transfusion volume per total of patients, the use of ANH dramatically reduced the volume of FFP transfusion per total of patients (32.00 ± 114.46 mL vs 115.71 ± 181.00 mL, *P* = 0.033, Table 3).

For the entire cohort, the requirement of allogeneic RBC transfusion was significantly associated with the length of surgery (306.6 ± 82.7 min vs 220.8 ± 64.5 min, *P* < 0.001, Table 4), estimated blood loss (910.0 ± 333.9 mL vs 300.0 ± 152.6 mL, *P* < 0.001, Table 4), and tumor size (5.9 ± 1.9 mm vs 4.6 ± 2.1 mm, *P* = 0.034, Table 4). Age, gender, body mass index, preoperative hemoglobin level, recurrence tumor, and deep location of tumor did not impact the perioperative allogeneic blood transfusion rate (Table 4).

**Table 4:** Risks associated with allogeneic red cell transfusion rate

	**Allogeneic red cell transfusion (*n* = 15)**	**None (*n* = 45)**	***P* value**
Age (years)	49.7 ± 15.7	48.6 ± 14.6	0.799
Male, %	10 (66.7%)	28 (62.2%)	0.757
Body mass index	24.1 ± 3.2	23.0 ± 3.2	0.270
Recurrence glioblastoma, %	3 (20%)	5 (11.1%)	0.661
Deep location, %	8 (53.3%)	17 (37.8%)	0.290
Tumor size (mm)	5.9 ± 1.9	4.6 ± 2.1	0.034
Preoperative hemoglobin (g/dL)	13.3 ± 2.4	13.3 ± 1.7	0.959
Estimated blood loss (mL)	910.0 ± 333.9	300.0 ± 152.6	< 0.001
Length of surgery (min)	306.6 ± 82.7	220.8 ± 64.5	< 0.001

Data are mean ± SD or *n* (%). *P* values were calculated by Student’s *t* test, Mann–Whitney *U* test, chi-square test, or Fisher’s exact test, as appropriate

## Discussion

Major glioblastoma resection would cause massive blood loss and often require perioperative blood transfusion. Blood shortage should be considered when arranging glioblastoma surgery. Allogeneic blood transfusion has some life-threatening risks, including immunomodulatory effect, coagulopathy, transfusion reaction, and mis-transfusion [11, 17]. Minimizing allogeneic blood transfusion will benefit patients and relieve the blood shortage.

ANH is considered as a safe and effective blood conservation technique [18]. First, unlike preoperative autologous blood donation, it is not needed to collect autologous blood repeatedly and save it for several days before surgery in the ANH group [8]. Second, intraoperative cell salvage is another commonly used blood conservation technique. However, its use in malignant cancer resection remains controversial, since it may cause cancer metastasis [19]. Till now, several studies have demonstrated the safety and efficiency of ANH in reducing the allogeneic blood transfusion rate [8, 20, 21]. However, the role of ANH in glioblastoma surgery remains unknown.

This study revealed that ANH was safe and effective in reducing the need for perioperative allogeneic blood transfusion in glioblastoma surgery. Two previous studies suggested that ANH was safe and did not influence the cerebral oxygenation parameters in brain tumor resection [22, 23]. However, these studies did not demonstrate the specific types of brain tumors, and more importantly, the role of ANH in glioblastoma surgery was not clarified. Mild volume ANH was used in this study, and this was because ① the average estimated blood loss in our previous experience was about 450 mL, which was consistent with previous reports (200–600 mL) [3, 24], and ② severe ANH may cause brain damage [25]. Consistent with previous reports [20, 21, 26], our results showed that ANH dramatically reduced perioperative allogeneic RBC transfusion in glioblastoma surgery. Additionally, the rate of FFP transfusion was significantly decreased, indicating that ANH was effective in conserving coagulation factors. The fact that blood loss was less in the ANH group may be due to the potential conserving effect of coagulation factors by ANH. Interestingly, the level of postoperative serum hemoglobin in the ANH patients returned to normal more quickly and was significantly higher.

With regards to safety, ANH did not increase the spectrum or incidence of complications as compared with those in the control group, and the complication rate was consistent with previous reports [3, 16]. Pulmonary infection seemed more common in the ANH group. However, the pulmonary infection rate has no difference as compared with the previous report (7.1–23.3%) [3]. For the patients with pulmonary infection in the ANH group, two were associated with postoperative aspiration-related pneumonia, one was caused by long-term tracheal intubation after secondary operation for postoperative hematoma, and one was suggested to be correlated with preoperative chronic pneumonia. None was considered as ANH-related as re-evaluated by two investigators. Actually, ANH reduced pulmonary infection rate in cardiac surgery and can be safely used in acute lung injury patients [13, 27]. Length of surgery, hospital length of stay, and discharge outcome have no difference between the two groups.

Several limitations should be considered. First, the sample was too small to assess the difference, and a risk estimation based on only 60 enrolled patients was accompanied by a wide variation; Further large cohort study is warranted. Second, the external validity of this study is affected by its single institution scope and its retrospective nature. Third, the acute reduction of serum hemoglobin induced by ANH may cause harm to patients. However, mild ANH basically does not affect the blood flow stability [25].

## Conclusions

We provided the first evidence that the use of mild ANH was safe and effective in reducing perioperative allogeneic blood transfusion in glioblastoma surgery. Further prospective large cohort study with long-term follow-up is warranted to confirm these findings.

## Data Availability

Data will be made available on request.

## Abbreviations

ANH: Acute normovolemic hemodilution
FFP: Fresh frozen plasma
KPS: Karnofsky Performance Scale
RBC: Red blood cell
SD: Standard deviation
